# Cognitive Dysfunctions Measured with the MCCB in Deficit and Non-Deficit Schizophrenia

**DOI:** 10.3390/jcm12062257

**Published:** 2023-03-14

**Authors:** Piotr Plichta, Ernest Tyburski, Maksymilian Bielecki, Monika Mak, Jolanta Kucharska-Mazur, Piotr Podwalski, Katarzyna Rek-Owodziń, Katarzyna Waszczuk, Leszek Sagan, Anna Michalczyk, Błażej Misiak, Jerzy Samochowiec

**Affiliations:** 1 Department of Health Psychology, Pomeranian Medical University in Szczecin, 71-457 Szczecin, Poland; 2 Department of Psychiatry, Pomeranian Medical University in Szczecin, 71-457 Szczecin, Poland; 3 Department of Neurosurgery, Pomeranian Medical University in Szczecin, 71-252 Szczecin, Poland; 4 Department of Psychiatry, Wroclaw Medical University, 50-367 Wroclaw, Poland

**Keywords:** schizophrenia, cognitive functions, psychopathology, deficit schizophrenia, non-deficit schizophrenia, MCCB, PANSS

## Abstract

This study compared cognitive domains between deficit schizophrenia (DS) and non-deficit schizophrenia (NDS) patients and healthy controls (HC), analyzing relationships between psychopathological dimensions and cognitive domains. A total of 29 DS patients, 45 NDS patients, and 39 HC subjects participated. Cognitive domains were measured using the Measurement and Treatment Research to Improve Cognition in Schizophrenia Battery. Psychopathological symptoms were evaluated with the Positive and Negative Syndrome Scale. Clinical groups performed poorer than HC groups in regards to speed of processing, attention/vigilance, working memory, verbal and visual learning and memory, reasoning and problem solving, and social cognition. DS patients scored poorer than NDS patients in terms of all cognitive domains and the overall score, except for reasoning and problem solving. Positive, negative, disorganization, and resistance symptoms were related to cognitive functions only in NDS patients. Our findings suggest that the MCCB battery is sensitive to detecting cognitive dysfunctions in both deficit and non-deficit schizophrenia.

## 1. Introduction

Cognitive impairment is observed across all stages of schizophrenia, from the premorbid period, through the high-risk state, to first episode psychosis, and ultimately, to the chronic course of the disease. The emerging deficits manifest to varying extents, depending on the phase of the illness. They primarily involve attention, working memory, executive functions, verbal and visual memory, social cognition, language, processing speed, and verbal fluency [1,2,3]. However, schizophrenia is characterized by extreme heterogeneity and marked clinical variance in psychopathological presentation, underlying biological correlates, functional outcomes, and response to treatment, which hinder the possibility of fully understanding the nature of specific or general cognitive dysfunctions. As a result, researchers have systematically parsed the symptomatology of schizophrenia into more homogeneous diagnostic categories, such as paranoid and non-paranoid, positive and negative, or deficit and non-deficit subtypes [4]. 

First identified by Carpenter et al., deficit schizophrenia (DS) with predominant negative symptoms is characterized by the presence of greater dysfunction and a long duration of symptomatology [5]. The primary features of this subtype are, among others, social withdrawal, poverty of speech, limited content of verbal expression, apathy, and blunted affect. Alongside the manifested negative deficits, the basic symptoms include: distortion of reality, disorganization, and cognitive impairment. Compared to the non-deficit subtype, deficit schizophrenia is associated with greater functional and structural disorders of the brain, which can be related to more severe cognitive dysfunctions [6,7].

Most studies to date demonstrate that patients with deficit schizophrenia manifest greater impairment in the performance of neuropsychological tasks. However, their results do not clearly indicate whether the observed dysfunction reflects a general or specific cognitive deficit [8]. Some light on this unclear matter is shed by the results of two meta-analyses [3,9], suggesting more general cognitive impairments in deficit compared to non-deficit schizophrenia. Notwithstanding, the authors propose that some patients with deficit schizophrenia may in fact manifest a differential pattern of neuropsychological impairment, which could be considerably more complicated than previously thought and which warrants a more sophisticated and rigorous examination of the cognitive dysfunctions underlying the deficit syndrome with the use of more extensive batteries of tests.

Although there is evidence of greater executive dysfunctions and reduced information processing speed in deficit schizophrenia [10,11,12,13,14,15,16], research findings concerning other cognitive domains are still markedly inconsistent. Some reports suggest more severe impairment within simple attention and working memory [11,17] or verbal and visual memory in this patient population [18,19]. Others show no differences between various patient groups in regards to mental flexibility, attention, and verbal memory [20], verbal working memory and verbal learning [21], or attention and working memory [22].

Of note, there seem to be certain gaps in previous studies that need to be addressed. For example, visual memory but not visual learning (except [18]) tends to be examined using a single trial only [19,23,24] in several trials, such as in the Brief Visuospatial Memory Test-Revised [25]. Likewise, there is a tendency to measure working memory using the Digit Span test [8,17,19,21,22,24,26], which is an easier task compared to the Letter-Number Sequencing test, which involves greater reliance on the central executive system [27]. In addition, only a few studies explored visuospatial working memory in deficit schizophrenia with the use of the Spatial Span or other related tasks [3]. What is more, to the best of our knowledge, previous studies did not use the Neuropsychological Assessment Battery—Mazes to measure reasoning and planning in deficit schizophrenia (except for Fervaha et al. [27], but they used the more simple Mazes from Revised Wechsler Intelligence Scale for Children). There are also some uncertainties regarding differences in social cognition between various patient groups. Most previous studies used a simple task to measure facial emotion recognition [12,20,21,23,27,28,29,30], providing limited information on how deficit schizophrenia affects performance in the Mayer–Salovey–Caruso Emotional Intelligence Test, which is a more complex task measuring the theory of the mind [31]. Thus, the answer to this emerging need to assess cognitive impairment underlying deficit schizophrenia in a more comprehensive manner might lie in the use of a structured test battery that could offer an extensive approach to the cognitive domains that are relevant to this subtype.

Another important issue is the relationship between psychopathological symptoms and cognitive impairment in different subtypes of schizophrenia. The available literature proposes different views on these links [32]. Nevertheless, various meta-analyses suggest a clear relationship between negative, positive, and disorganization symptoms and cognitive functioning in schizophrenia [33,34,35]. Seemingly, however, the analysis of the links between psychopathological symptoms and cognitive deficits in deficit schizophrenia has been widely neglected (c.f. [3,9]). Even though Yu et al. [26] found a relationship between negative symptoms and cognitive function in both deficit and non-deficit schizophrenia, these links differed within different cognitive domains. What is more, negative symptoms and cognitive functions were found to be influenced by age in the deficit variant and by age, education, and illness duration in the non-deficit type. Chen et al. [23] reported negative correlations between: (i) general psychopathology symptoms and verbal memory in first-episode drug-naive schizophrenia with the deficit syndrome; and (ii) positive symptoms for verbal and visual memory in the non-deficit syndrome. In turn, Tang et al. [30] demonstrated a negative relationship between positive, disorganization, and negative symptoms and certain emotions in the context of facial emotion recognition in non-deficit schizophrenia, but not in deficit schizophrenia.

To address the above limitations, we compared different cognitive domains (speed of processing, attention/vigilance, working memory, verbal and visual learning memory, reasoning and problem solving, and social cognition) and the general score of the Measurement and Treatment Research to Improve Cognition in Schizophrenia Battery (MCCB) [36,37] between deficit and non-deficit schizophrenia patients, along with the healthy controls. While the relationship between psychopathological symptoms and cognitive domains in schizophrenia has been the subject of many studies, it remains underexplored in deficit schizophrenia. A meta-analysis of the Positive and Negative Syndrome Scale (PANSS [38]) suggests that it has a five-factor structure—positive symptoms, negative symptoms, disorganization, affect, and resistance—that can be extremely useful for assessing the severity of symptoms; however, it remains underused in studies on cognitive functioning in DS. Thus, we estimated the relationships between the five PANSS factors and cognitive domains in both clinical groups. Based on previous findings, we hypothesized that deficit schizophrenia patients would manifest greater cognitive impairment and overall scores relative to non-deficit schizophrenia and the controls. Moreover, we assumed that a complex relationship would emerge between different psychopathological dimensions and cognitive domains, and that it would differ in both clinical groups.

## 2. Materials and Methods

### 2.1. Participants

This study included 74 patients diagnosed with schizophrenia based on the International Statistical Classification of Diseases and Related Health Problems (ICD-10 [39]) and the Mini-International Neuropsychiatric Interview (MINI) [40]), and 39 healthy participants (without mental or neurological disorders). A total of 29 patients were further diagnosed with DS (based on the criteria proposed by Carpenter et al. [5]; for more details concerning recruitment and inclusion criteria, see [41]), and 45 with NDS. The clinical group was recruited in cooperation with the Department of Psychiatry of the Pomeranian Medical University and outpatient mental health clinics in Szczecin, Poland. Healthy controls were recruited through information spread by students and staff of the local universities. The inclusion criteria in the clinical group were: diagnosis of schizophrenia, disease duration of ≥10 years, age 30–55 years, and informed consent to participate in the study. In the control group they were: age 30–55 years and informed consent to participate in the study. The exclusion criteria were: mental illness other than schizophrenia or other neurological disorders, alcohol or substance use disorder, chronic somatic comorbidity that may affect cognitive functioning (e.g., severe diseases of parenchymal organs and/or cancer), and a history of head trauma. All participants underwent a psychological and psychiatric examination. The former included assessment of intellectual and cognitive functioning, while the latter comprised a detailed clinical history, measurement of neuropsychiatric symptoms, and an overall assessment of health. All patients provided written consent to participate in the study. The study protocol was approved by the local bioethics committee.

### 2.2. Neuropsychological Assessment

The Polish version of the Measurement and Treatment Research to Improve Cognition in Schizophrenia Battery (MCCB [36,37]) was used to measure different cognitive domains. This battery includes several subtests to assess:

- Speed of processing—Trail Making Test (TMT: Part A), Brief Assessment of Cognition in Schizophrenia (BACS), and the Category Fluency Test: Animal Naming (CF);
- Attention/vigilance—Continuous Performance Test—Identical Pairs (CPT-IP);
- Working memory—Letter-Number Span (LNS) and Wechsler Memory Scale-III (WMS III);
- Verbal learning and memory—Hopkins Verbal Learning Test-Revised (HVLT-R);
- Visual learning and memory—Brief Visuospatial Memory Test-Revised (BVMT-R);
- Reasoning and problem solving—Neuropsychological Assessment Battery (NAB): Mazes.
- Social cognition—Mayer–Salovey–Caruso Emotional Intelligence Test (MSCEIT).

Scores for different cognitive domains and overall scores were calculated using the mean of the nine demographically corrected T-scores using the computer application of MCCB [37].

Moreover, general intellectual ability as indirect premorbid IQ was assessed with the Vocabulary and Picture Completion measures of the Wechsler Adult Intelligence Scale—Revised, a standardized tool to measure adult general intelligence [42]. Both subtests were often used to measure indirect (case-control studies) and direct (longitudinal studies) premorbid IQ in schizophrenia (Khandaker et al. [43]) and previous studies have demonstrated strong links between both subtests and full IQ [44,45] in schizophrenia patients. Based on numerous recommendations [46,47,48,49,50], we selected the Vocabulary subtest as a measure of indirect premorbid crystalized IQ and Picture Completion as a measure of indirect premorbid fluid IQ.

### 2.3. Clinical Assessment

We used the Positive and Negative Syndrome Scale (PANSS) [51,52] to measure psychopathological symptoms in DS and NDS patients, adopting the five factor structure identified by Shafer and Dazzi [38], including positive and negative, disorganization, affect, and resistance symptoms. We also used the Polish versions of the Brief Negative Symptom Scale (BNSS) [53] and the Self-Evaluation of Negative Symptoms (SNS) [54] to measure deficit symptoms. The BNSS is a reliable tool composed of a total of 13 items to assess five domains of negative symptoms, i.e., anhedonia, asociality, avolition, blunted affect, and alogia. Each item is rated on a seven-point severity scale, from 0 (symptom absent) to 6 (severe). The SNS consists of 20 self-report items, evaluating 5 subdomains (social withdrawal, diminished emotional range, avolition, anhedonia, and alogia) over the course of the previous week. The Global Assessment of Functioning (GAF) [55] was used to assess the severity of schizophrenia and its impact on patients’ functioning. 

### 2.4. Statistical Analysis

Analysis of the results was performed in IBM SPSS 28 (IBM Corp., Redmont, VA, USA). Continuous variables were described in terms of means (*M*) and standard deviations (*SD*). The Shapiro–Wilk test and skewness and kurtosis values were used to check the normality of the distributions. We took skewness and kurtosis values ranging from −2 to +2 as indicating normal distribution [56]. Years of education, age, premorbid crystalized IQ (WAIS-R-IV Vocabulary), and attention/vigilance, working memory, reasoning and problem solving, social cognition, and overall score on the MCCB were normally distributed in all groups. In the patient groups, global functioning (GAF), chlorpromazine equivalent, and duration of illness showed normal distributions. The following were not normally distributed: exacerbation, results for all PANSS factors, premorbid IQ (WAIS-R-IV Picture Completion), negative symptoms on the BNSS and SNS, and scores on three cognitive indices on the MCCB (speed of processing, verbal learning and memory, and visual learning and memory) were non-normally distributed. Before performing further analyses, we therefore logarithmically transformed exacerbation and negative symptoms on SNS, and Box–Cox transformed the other variables to achieve normal distributions [57]. The Student’s *t*-test was used to investigate differences between the two clinical groups (clinical factors and psychopathological symptoms). An analysis of variance (ANOVA) was used to examine differences between the three groups in different aspects of cognitive domains and overall MCCB score. The Bonferroni post hoc test was used for comparisons between groups (for parametric tests). To calculate the magnitudes of effect sizes of differences between groups, Cohen’s *d* and ɳ^2^ (continuous variables) and Cramér’s *V* (categorical variables) were used [58]. Furthermore, Pearson’s *r* correlation coefficients were used to assess the relationships between psychopathological symptoms and different cognitive domains and overall score in the clinical groups. G*Power software was used to estimate the sensitivity analysis for ANOVA [59]. According to its results, ANOVA with 113 participants across three groups would be sensitive to effects of ɳ^2^ = 0.12 with 95% power (*p* = 0.05). This means that our study would not be able to reliably detect effects smaller than ɳ^2^ = 0.12. An alpha value of 0.05 was used for all analyses.

## 3. Results

### 3.1. Demographic, Psychological and Clinical Characteristics

**Figure 1:**
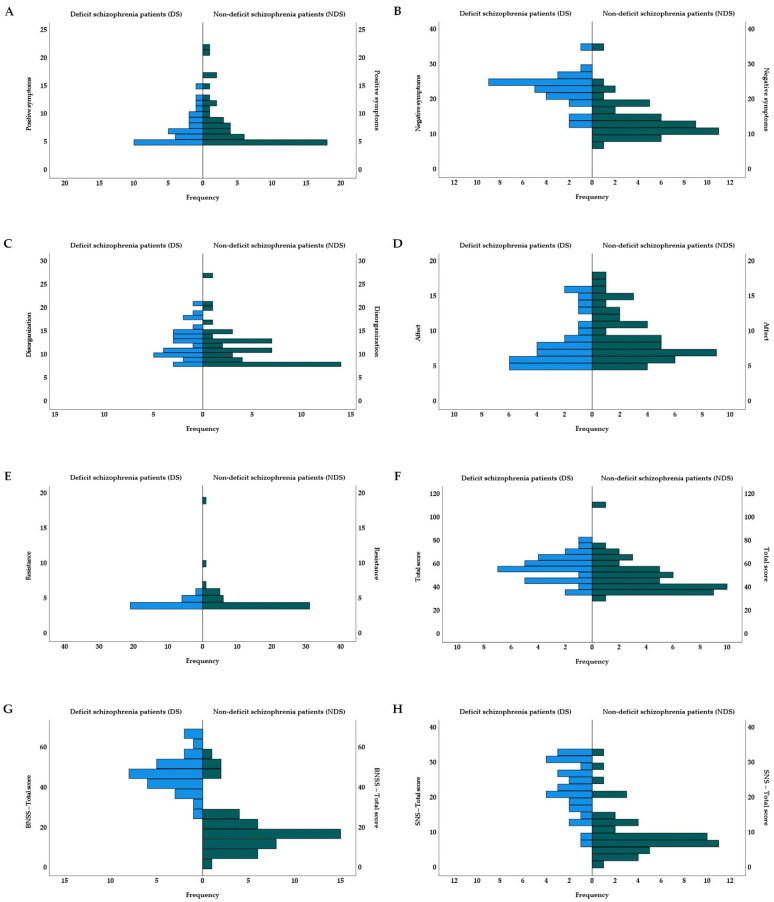
Distributions of the samples on psychopathological dimensions from two clinical groups—raw scores (PANSS: (**A**) = positive symptoms; (**B**) = negative symptoms; (**C**) = disorganization; (**D**) = affect; (**E**) = resistance; (**F**) = total score; (**G**) = BNSS—total score, and (**H**) = SNS—total score). BNSS = Brief Negative Symptom Scale. PANSS = Positive and Negative Syndrome Scale. SNS = Self-Evaluation of Negative Symptoms.

Table 1 shows the demographic, psychological, and clinical characteristics of the groups. While there were no significant differences in age, the groups differed significantly in years of education (*p* = 0.020), sex (*p* = 0.011), premorbid crystalized IQ measured by WAIS-R-IV Vocabulary (*p* < 0.001), and premorbid fluid IQ measured by WAIS-R-IV Picture Completion (*p* < 0.001). There were more males than females in the DS group, and post hoc analyses revealed that DS patients had fewer years of education than the HC group (*p* = 0.029), and they showed lower fluid IQ than did the NDS and HC groups (*p* = 0.008 and *p* < 0.001), as well as lower crystalized IQ than NDS and HC (both: *p* < 0.001). Similarly, NDS patients had lower fluid and crystalized IQ than HC (both: *p* < 0.001). After Holm–Bonferroni *p*-value correction, DS patients showed more negative symptoms and a larger total score on PANSS (*p* < 0.001 and *p* = 0.005), more negative symptoms on BNSS (*p* < 0.001), and more negative symptoms on SNS (*p* < 0.001) than NDS patients. There were no significant differences between clinical groups in terms of antipsychotic medications, chlorpromazine equivalent, duration of illness, exacerbation, global functioning in GAF, or other psychopathological symptoms on PANSS (positive symptoms, disorganization, affect, or resistance). Additionally, the distributions of the samples regarding psychopathological dimensions from two clinical groups are presented in Figure 1.

### 3.2. Differences in Cognitive Domains

**Figure 2:**
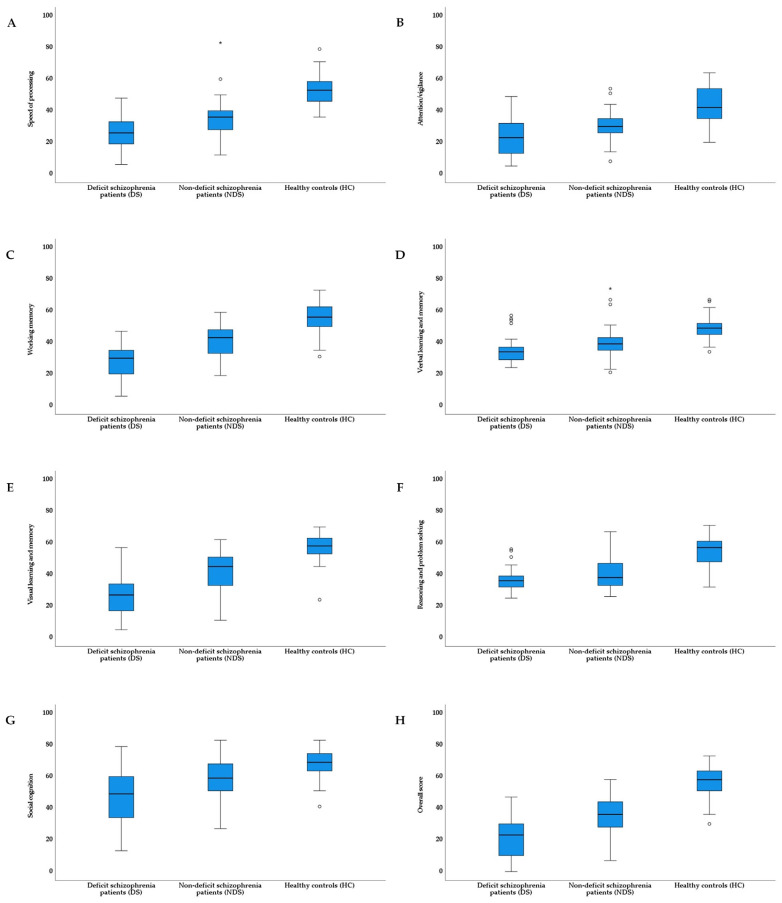
Comparison of cognitive functions between participants from three groups—untransformed T-scores (MCCB: (**A**) = speed of processing; (**B**) = attention/vigilance; (**C**) = working memory; (**D**) = verbal learning and memory; (**E**) = visual learning and memory; (**F**) = reasoning and problem solving; (**G**) = social cognition, and (**H**) = overall score). In all box plots, the bottom end of the box designates the first quartile, a line within the box indicates the median, and the top end of the box shows the third quartile. * indicate values 1.5 times the interquartile range below the first quartile and above the third quartile. Crosses represent average values. Circles designate individual observations. MCCB = Measurement and Treatment Research to Improve Cognition in Schizophrenia.

As shown in Table 2 (T-scores before and after transformation) and Figure 2 (T-scores before transformation), the three groups differed significantly in all cognitive domains, in speed of processing (*p* < 0.001), attention/vigilance (*p* < 0.001), working memory (*p* < 0.001), verbal learning and memory (*p* < 0.001), visual learning and memory (*p* < 0.001), reasoning and problem solving (*p* < 0.001), social cognition (*p* < 0.001), and overall score (*p* < 0.001). Post hoc analysis indicated that DS patients had lower scores on speed of processing (both: *p* < 0.001), attention/vigilance (*p* = 0.031 and *p* < 0.001), working memory (both: *p* < 0.001), verbal learning and memory (*p* = 0.019 and *p* < 0.001), visual learning and memory (both: *p* < 0.001), social cognition (*p* = 0.005 and *p* < 0.001), and overall score (both: *p* < 0.001) than both the NDS and HC groups, as well as lower scores for reasoning and problem solving (*p* < 0.001) than the HC group. Moreover, NDS patients exhibited lower scores than the HC for all cognitive domains (*p* < 0.001) and social cognition (*p* = 0.002). Additionally, distributions of the samples regarding cognitive functions from three groups are presented in Figure S1 in Supplementary Materials.

### 3.3. Relationships between Psychopathological Dimensions and Cognitive Domains

As can be seen in Table 3, there were no significant correlations between psychopathological symptoms and cognitive domains in DS patients. However, in NDS patients: positive symptoms correlated negatively with speed of processing (*r* = −0.33; *p* = 0.027), reasoning and problem solving (*r* = −0.31; *p* = 0.040), and overall score (*r* = −0.41; *p* = 0.005); negative symptoms correlated negatively with speed of processing (*r* = −0.30; *p* = 0.048); disorganization correlated negatively with speed of processing (*r* = −0.41; *p* = 0.005), reasoning and problem solving (*r* = −0.39; *p* = 0.008), and overall score (*r* = −0.35; *p* = 0.019); and resistance correlated negatively with speed of processing (*r* = −0.46; *p* = 0.001). Correlation coefficients were not corrected.

## 4. Discussion

In this study, we examined differences in cognitive performance based on the use of the MCCB patients with deficit schizophrenia (DS) and non-deficit schizophrenia (NDS), as well as healthy controls. Moreover, we investigated the relationship between five psychopathological dimensions and cognitive domains.

Our results suggest greater cognitive deficits in DS patients compared to NDS patients in all examined cognitive domains, except reasoning and problem solving. They can, therefore, especially alongside qualitative analysis of distributions of cognitive domains in the clinical groups and healthy controls, further support the construct validity of the DS versus NDS distinction. In their meta-analysis, Bora et al. [3] showed similar results, i.e., significant differences between patient groups in processing speed, attention, working memory, verbal and visual memory, and social cognition. Moreover, we demonstrated greater general cognitive dysfunctions in DS patients as reflected by overall scores on the MCCB. Some authors suggest that DS patients manifest a general rather than specific cognitive dysfunction [8]. However, the absence of inter-group differences in reasoning and problem solving may mean that there are some cognitive domains that are less affected in deficit schizophrenia. Admittedly, the MCCB was not designed to measure executive functions, but reasoning and problem solving as measured by the Neuropsychological Assessment Battery—Mazes is also a measure of planning skills, which some researchers classify as executive functions [61]. It has been shown that in patients with DS, these functions are usually impaired to a greater extent, but not all studies corroborate these observations [16]. In earlier studies, tests such as the Wisconsin Card Sorting Test, the Trail Making Test, or the Stroop Test were mainly used to assess executive functions. We found no studies that used the NAB—Mazes, so it is difficult to compare our results against those of other authors. However, Fervaha et al. [27] found significant group differences, but a small effect size, using Mazes from WISC-R. It seems that further research is needed to determine whether some aspects of executive functions, such as planning, are actually more or less affected by the deficit syndrome.

Our results suggest greater impairments in deficit schizophrenia in terms of working memory measured by two tests (Letter-Number Span and Wechsler Memory Scale-III) included in the MCCB, which are designed to assess pattern-spatial memory and verbal memory, with a test that burdens the central executive system to a greater extent. Similar results have been shown in previous studies using simpler tests to assess verbal working memory and the SS test for visuospatial working memory [11,27]. We also found greater deficits in verbal and non-verbal learning in people with DS, in line with the results of previous studies using the same tests (Hopkins Verbal Learning Test-Revised and Brief Visuospatial Memory Test-Revised [27]). Our results therefore demonstrate that people with DS are affected not only by memory deficits measured by single task trials, but also by more complex learning tests involving a series of repeated learning attempts using verbal and visual material. Previous studies showed greater deficits in social cognition in DS as measured by simple facial emotion recognition tests. Our study further suggests that DS patients may have deficits in theory of mind as assessed by a more complex task in which subjects must interpret a series of stories and empathize with the thinking of others.

Moreover, we showed that both clinical groups exhibited significantly lower scores in all cognitive domains and the overall score compared to healthy individuals, which means that both DS and NDS patients have general cognitive impairments. Previous studies using the MCCB in schizophrenia have repeatedly demonstrated the presence of such deficits [62]. Our study also concludes that the MCCB is sensitive to cognitive deficits, both in various cognitive domains and in general cognitive functioning, in deficit schizophrenia. Due to the fact that DS patients showed greater functional and structural abnormalities of the brain compared to NDS patients and healthy people [6,7], it would be worthwhile in the future to determine the relationship between the volume of various brain structures and the integrity of the white matter of the bundles connecting cognitively important structures with the results in individual cognitive domains assessed by the MCCB. Such research could shed more light on the role of changes in the brain, which in deficit schizophrenia, may be one of the potential neurobiological correlates of cognitive dysfunction, and which, according to both neurodevelopmental and neurodegenerative theories, most likely contribute to the poorer functioning of patients.

We did not observe a significant relationship between psychopathological symptoms and cognitive functions in DS patients, which may very likely be attributed to a small sample size. However, only few previous studies analyzed the relationship between psychopathological symptoms and cognitive functions in deficit schizophrenia. Their results were also inconsistent, as some showed an association between negative symptoms and attention [26] or global symptoms and verbal memory [23]. In turn, Tang et al. [30] did not show any relationship between psychopathological symptoms and facial emotion recognition. Perhaps the inter-group relationships between deficit schizophrenia and cognitive disorders (based on comparisons of different schizophrenia patient populations) are not the same as intra-group relationships (based on correlation analysis). A certain explanation for the lack of such a correlation in DS patients may be that the PANSS does not fully measure the symptoms characteristic of the deficit syndrome, i.e., the negative symptoms that initially occur. It seems that further studies are needed in which other scales should be used to assess deficit symptoms in the context of their relationship with cognitive functions in DS patients. On the other hand, a relationship between some psychopathological symptoms and cognitive functions emerged in non-deficit schizophrenia, where positive, negative, and resistance symptoms were negatively associated with speed of processing, and positive and disorganization symptoms with reasoning and problem solving. Similar results were provided by previous studies [32,33,34]. However, due to the fact that the non-deficit schizophrenia set is a very heterogeneous group, it is difficult to clearly explain the relationships with some cognitive domains. Several theoretical models have been proposed in the literature [32], explaining, for example, the relationship between negative symptoms and cognitive functions, but our study does not allow for the verification of such models. Therefore, future studies are needed in which methodological solutions will allow for a better explanation of the relationship between psychopathology and cognitive disorders in schizophrenia.

Moreover, our results suggest psychopathological presentation between DS and NDS patients. Indeed, differences in psychopathology emerged within the negative dimension of the disease spectrum measured by the three administered scales (PANNS, BNSS, and SNS). Thus, in line with other studies on psychopathological manifestation in DS [4,15,63], our findings, combined with qualitative analysis of distributions of psychopathological dimensions, support the postulated construct validity of the DS-NDS distinction. Our sample did not differ significantly in terms of age, antipsychotic medications, chlorpromazine equivalent, duration of illness, or exacerbation. Additionally, no inter-group differences emerged in terms of general functioning as measured by the GAF, which is inconsistent with other reports [63] and may result from a small sample size. The absence of differences in psychopathology in other dimensions is likely attributable to our study design. In addition, since they were not in acute psychosis, the observed negative symptoms were not due to other psychopathology, but reflected the general DS criteria.

This study has some potential limitations. The first limitation is that the group of respondents was relatively small. The small sample of patients limits the generalizability of the conclusions. It would be of great scientific value to increase the number of participants in the study. The second limitation was that the proportion of females and males in each patient group was not homogeneous (there were more male DS patients). There are some findings suggesting that female hormones benefit the brain areas involved in cognitive function [64]. This may be an alternative explanation for why NDS patients performed better on cognitive tasks than DS patients. However, it is noted that sex is one of the risk factors for deficit syndrome in schizophrenia. Previous studies show that more males than females have a diagnosis of deficit schizophrenia [3,4]. The third limitation is that there were significant differences in patient groups in fluid and crystalized intelligence. Some previous studies suggest that DS patients manifest poorer intellectual functioning than NDS patients, which is probably an element characterizing this clinical population. Moreover, we used only two subtests from the WAIS-R to measure indirect premorbid IQ. Some researchers recommend cautious use of abbreviated forms when it is necessary to estimate the factor index scores, and many data suggest that a statistical search for a “best” short form is largely futile [50]. Thus, short forms should be selected on the basis of their efficiency in providing the required information [47]. The fourth limitation concerns the inclusion criteria for the patient groups: illness duration of ≥10 years and age of 30–50 years. We sought to compare DS and NDS patients as a more homogenous group. However, as this may (significantly) restrict our ability to generalize our results to the entire schizophrenia population, our results should be interpreted with great caution. Our study is cross-sectional and as such, cannot control for potential cohort effects. Longitudinal studies have many advantages for describing differences between clinical trials because this type of study design allows for the observation of changes over time in the same participants. 

## 5. Conclusions

Our study found cognitive impairments in patients with both deficit and non-deficit schizophrenia. The severity of these impairments was greater in deficit schizophrenia patients, except for reasoning and problem-solving. Our findings suggest that the MCCB battery is sensitive for detecting cognitive dysfunctions, not only in non-deficit schizophrenia but also in deficit schizophrenia. Psychopathological dimensions seem to play a significant role in the cognitive performance of non-deficit schizophrenia patients only, but their relationship seems to be complex. However, these conclusions have limited generalizability, as the sample size was small. Finally, our results suggest that non-deficit schizophrenia is a heterogeneous concept, and some patients with non-deficit schizophrenia may present persistent negative symptoms which may not necessarily be considered as primary.

## Figures and Tables

**Table 1:** Demographic, psychological, and clinical characteristics of all participants.

	Deficit Schizophrenia Patients (DS) (*n* = 29)	Non-Deficit Schizophrenia Patients (NDS) (*n* = 45)	Healthy Controls (HC) (*n* = 39)	*F*/*χ^2^*/*t*	ɳ^2^/*V*/*d*
Age: *M* (*SD*)	38.59 (6.17)	39.16 (7.21)	37.08 (7.94)	0.90 ^c^	0.02 ^f^
Years of education: *M* (*SD*)	12.66 (3.24) ^i^ *	13.53 (2.64)	14.59 (2.62)	4.06 ^c^ *	0.07 ^f^
Sex: female/male	7/22	24/21	23/16	9.01 ^d^ *	0.28 ^g^
Premorbid IQ in WAIS-R-IV:					
Picture Completion: *M* (*SD*)	17.86 (7.60)/20.52 (13.35) ^b,i,^ ***^, j^ *	22.56 (6.13)/29.53 (13.24) ^b,k^ ***	29.62 (3.63)/47.46 (10.34) ^b^	43.27 ^c^ ***	0.44 ^f^
Vocabulary: *M* (*SD*)	33.97 (14.47) ^) i^ ***^, j^ **	43.40 (10.18) ^k^ ***	56.18 (6.55)	38.81 ^c^ ***	0.41 ^f^
Antipsychotic medications:					
Atypical: *n* (%)	20 (68.97)	29 (64.44)	-	2.09 ^d^	0.17 ^g^
Atypical and typical: *n* (%)	8 (27.58)	12 (26.67)	-		
Typical: *n* (%)	0 (0.00)	3 (6.67)	-		
No medications: *n* (%)	1 (3.45)	1 (2.22)	-		
Chlorpromazine equivalent (mg): *M* (*SD*)	695.86 (311.57)	644.04 (309.71)	-	0.71 ^e^	0.17 ^h^
Duration of illness: *M* (*SD*)	16.97 (5.73)	14.00 (5.14)	-	2.32 ^e^	0.55 ^h^
Exacerbation: *M* (*SD*)	5.69 (2.44)/1.64 (0.48) ^a^	6.49 (5.01)/1.65 (0.64) ^a^	-	−0.11 ^e^	−0.03 ^h^
Global functioning in GAF: *M* (*SD*)	50.93 (14.34)	58.40 (14.21)	-	−2.20 ^e^	−0.52 ^h^
Psychopathological symptoms in PANSS:					
Positive symptoms: *M* (*SD*)	7.38 (2.73)/5.28 (0.06) ^b^	8.07 (4.37)/5.28 (0.06) ^b^	-	0.00 ^e^	0.00 ^h^
Negative symptoms: *M* (*SD*)	22.24 (4.66)/5.85 (0.01) ^b^	13.80 (5.19)/5.80 (0.03) ^b^	-	7.45 ^e^ ***	1.51 ^h^
Disorganization: *M* (*SD*)	12.62 (3.48)/5.36 (0.02) ^b^	11.42 (3.98)/5.34 (0.03) ^b^	-	1.93 ^e^	0.46 ^h^
Affect: *M* (*SD*)	8.24 (3.45)/5.29 (0.06) ^b^	9.29 (3.53)/5.31 (0.05) ^b^	-	−1.68 ^e^	−0.40 ^h^
Resistance: *M* (*SD*)	4.34 (0.61)/5.04 (0.04) ^b^	4.89 (2.43)/5.05 (0.06) ^b^	-	−1.07 ^e^	−0.23 ^h^
Total score: *M* (*SD*)	56.83 (11.17)/5.40 (0.01) ^b^	49.33 (14.68)/5.40 (0.01) ^b^	-	3.31 ^e^ *	0.73 ^h^
Negative symptoms in BNSS:					
Total score: *M* (*SD*)	47.07 (9.28)/2.63 (0.43) ^b^	20.07 (12.68)/1.27 (0.66) ^b^	-	9.87 ^e^ ***	2.35 ^h^
Negative symptoms in SNS:					
Total score: *M* (*SD*)	22.28 (7.38)/3.03 (0.41) ^a^	9.71 (6.89)/2.05 (0.70) ^a^	-	−7.63 ^e^ ***	1.63 ^h^

BNSS = Brief Negative Symptom Scale. GAF = Global Assessment of Functioning. PANSS = Positive and Negative Syndrome Scale. SNS = Self-Evaluation of Negative Symptoms. WAIS-R-IV = Wechsler Adult Intelligence Scale Revised Fourth Edition. Chlorpromazine equivalents were calculated based on a proposition by [60]. ^a^ Mean and standard deviation after logarithmic transformation. ^b^ Mean and standard deviation after Box–Cox transformation. ^c^ One-way analysis of variance *F* test. ^d^ Chi-squared test. ^e^ Student’s *t*-test. ^f^ Eta squared effect size: small (0.01–0.059), medium (0.06–0.139), large (0.14–1.00). ^g^ Cramer’s *V* effect size: small (0.10–0.19), medium (0.20–0.59), large (0.60–1.00). ^h^ Cohen’s *d* effect size: small (0.20–0.49), medium (0.50–0.79), large (0.80 <). All *p*-values for ANOVA. ^i^ DS patients vs. HC participants. ^j^ DS patients vs. NDS patients. ^k^ NDS patients vs. HC participants. * *p* < 0.05, ** *p* < 0.01, *** *p* < 0.001. (after Holm–Bonferroni *p*-value correction for Student’s *t*-test).

**Table 2:** Comparison of MCCB cognitive domains between all participants...

	Deficit Schizophrenia Patients (DS) (*n* = 29)	Non-Deficit Schizophrenia Patients (NDS) (*n* = 45)	Healthy Control (HC) (*n* = 39)	*F*	ɳ^2^
Speed of processing	25.93 (11.53)/3.19 (0.49) ^a,c^ ***^,d^ ***	34.62 (12.40)/3.51 (0.36) ^a,e^ ***	53.15 (9.66)/3.97 (0.18) ^a^	43.25 ***	0.44
Attention/vigilance	23.69 (11.58) ^c^ ***^,d^ *	30.22 (8.76) ^e^ ***	42.90 (11.55)	30.12 ***	0.35
Working memory	27.72 (11.40) ^c^ ***^,d^ ***	40.67 (10.95) ^e^ ***	54.51 (9.57)	53.66 ***	0.49
Verbal learning and memory	34.76 (9.24)/3.54 (0.24) ^a,c^ ***^,d^ *	40.09 (10.11)/3.68 (0.24) ^a,e^ ***	48.95 (7.50)/3.90 (0.15) ^a^	24.27 ***	0.31
Visual learning and memory	28.00 (13.78)/5.12 (4.56) ^b,c^ ***^,d^ ***	41.71 (12.65)/9.90 (5.05) ^b,e^ ***	56.90 (8.21)/17.08 (4.29) ^b^	57.23 ***	0.51
Reasoning and problem solving	37.24 (7.85) ^c^ ***	41.49 (11.15) ^e^ ***	54.44 (9.20)	30.50 ***	0.36
Social cognition	48.24 (16.42) ^c^ ***^,d^ **	58.00 (12.40) ^e^ **	67.87 (9.33)	20.20 ***	0.27
Overall score	20.31 (12.88) ^c^ ***^,d^ ***	34.87 (11.78) ^e^ ***	56.69 (9.13)	91.29 ***	0.62

MMCB = cognitive functioning was evaluated with Measurement and Treatment Research to Improve Cognition in Schizophrenia. *F* = Analysis of variance *F* test. ɳ^2^ = Eta squared effect size: small (0.01–0.059), medium (0.06–0.139), large (0.14–1.00). ^a^ Mean and standard deviation of T-scores after logarithmical transformation. ^b^ Mean and standard deviation of T-scores after Box–Cox transformation. ^c^ DS patients vs. HC participants. ^d^ DS patients vs. NDS patients. ^e^ NDS patients vs. HC participants. * *p* < 0.05, ** *p* < 0.01, *** *p* < 0.001.

**Table 3:** Relationship between psychopathological symptoms measured with the PANSS and cognitive domains measured with the MCCB in the patient groups.

Deficit Schizophrenia Patients (DS) (*n* = 29)								
	Speed of Processing	Attention/Vigilance	Working Memory	Verbal Learning and Memory	Visual Learning and Memory	Reasoning and Problem Solving	Social Cognition	Overall Score
	*r*	*r*	*r*	*r*	*r*	*r*	*r*	*r*
Positive symptoms	0.08	0.02	−0.10	0.13	−0.16	−0.34	0.01	−0.11
Negative symptoms	0.04	−0.13	−0.05	0.13	−0.26	0.01	−0.02	−0.08
Disorganization	−0.02	0.10	0.07	0.26	−0.04	−0.24	0.04	0.02
Affect	0.17	−0.29	0.17	0.08	−0.05	0.26	0.11	0.11
Resistance	−0.31	−0.01	−0.35	−0.07	−0.25	−0.30	−0.14	−0.34
Non-Deficit Schizophrenia Patients (NDS) (*n* = 45)								
	Speed of Processing	Attention/Vigilance	Working Memory	Verbal Learning and Memory	Visual Learning and Memory	Reasoning and Problem Solving	Social Cognition	Overall Score
	*r*	*r*	*r*	*r*	*r*	*r*	*r*	*r*
Positive symptoms	−0.33 *	−0.10	−0.25	−0.15	−0.28	−0.31 *	−0.25	−0.41 **
Negative symptoms	−0.30 *	−0.03	0.06	0.20	−0.04	−0.12	0.00	−0.10
Disorganization	−0.41 **	−0.03	−0.22	−0.08	−0.18	−0.39 **	−0.12	−0.35 *
Affect	−0.22	−0.10	−0.18	0.08	−0.11	−0.13	−0.05	−0.20
Resistance	−0.46 **	−0.01	−0.01	−0.13	−0.11	−0.27	−0.13	−0.25

MMCB = cognitive functioning was evaluated with Measurement and Treatment Research to Improve Cognition in Schizophrenia. PANSS = Positive and Negative Syndrome Scale. * *p* < 0.05, ** *p* < 0.01.

## Data Availability

Data and materials for the experiments reported here are available from the corresponding author on reasonable request.

## Supplementary Materials

The following supporting information can be downloaded at: https://www.mdpi.com/article/10.3390/jcm12062257/s1, FigureS1. Distributions of the samples on cognitive functions from three groups – untransformed T-scores (MCCB: (A) = speed of processing; (B) = attention/vigilance; (C) = working memory; (D) = verbal learning and memory; (E) = visual learning and memory; (F) = reasoning and problem solving; (G) = social cognition, and (H) = overall score). MCCB = Measurement and Treatment Research to Improve Cognition in Schizophrenia.

## References

[1] Fioravanti M., Bianchi V., Cinti M.E. (2012). Cognitive deficits in schizophrenia: An updated metanalysis of the scientific evidence. BMC Psychiatry.

[2] Liu Y., Wang G., Jin H., Lyu H., Liu Y., Guo W., Shi C., Meyers J., Wang J., Zhao J. (2019). Cognitive deficits in subjects at risk for psychosis, first-episode and chronic schizophrenia patients. Psychiatry Res..

[3] Bora E., Akdede B., Alptekin K. (2017). Neurocognitive impairment in deficit and non-deficit schizophrenia: A meta-analysis. Psychol. Med..

[4] Kirkpatrick B., Mucci A., Galderisi S. (2017). Primary, enduring negative symptoms: An update on research. Schizophr. Bull..

[5] Carpenter W.T., Heinrichs D.W., Wagman A.M. (1998). Deficit and nondeficit forms of schizophrenia: The concept. Am. J. Psychiatry.

[6] Voineskos A.N., Foussias G., Lerch J., Felsky D., Remington G., Rajji T.K., Lobaugh N., Pollock B.G., Mulsant B.H. (2013). Neuroimaging evidence for the deficit subtype of schizophrenia. JAMA Psychiatry.

[7] Podwalski P., Szczygieł K., Tyburski E., Sagan L., Misiak B., Samochowiec J. (2021). Magnetic resonance diffusion tensor imaging in psychiatry: A narrative review of its potential role in diagnosis. Pharmacol. Rep..

[8] Réthelyi J.M., Czobor P., Polgár P., Mersich B., Bálint S., Jekkel E., Magyar K., Mészáros A., Fábián A., Bitter I. (2012). General and domain-specific neurocognitive impairments in deficit and non-deficit schizophrenia. Eur. Arch. Psychiatry Clin. Neurosci..

[9] Cohen A.S., Saperstein A.M., Gold J.M., Kirkpatrick B., Carpenter W.T., Buchanan R.W. (2007). Neuropsychology of the deficit syndrome: New data and meta-analysis of findings to date. Schizophr. Bull..

[10] Brazo P., Marié R.M., Halbecq I., Benali K., Segard L., Delamillieure P., Langlois-Théry S., Van Der Elst A., Thibaut F., Petit M. (2002). Cognitive patterns in subtypes of schizophrenia. Eur. Psychiatry.

[11] Buchanan R.W., Strauss M.E., Kirkpatrick B., Holstein C., Breier A., Carpenter W.T. (1994). Neuropsychological impairments in deficit vs nondeficit forms of schizophrenia. Arch. Gen. Psychiatry.

[12] Horan W.P., Blanchard J.J. (2003). Neurocognitive, social, and emotional dysfunction in deficit syndrome schizophrenia. Schizophr. Res..

[13] Polgár P., Farkas M., Nagy O., Kelemen O., Réthelyi J., Bitter I., Myers C.E., Gluck M.A., Kéri S. (2008). How to find the way out from four rooms? The learning of “chaining” associations may shed light on the neuropsychology of the deficit syndrome of schizophrenia. Schizophr. Res..

[14] Polgár P., Réthelyi J.M., Bálint S., Komlósi S., Czobor P., Bitter I. (2010). Executive function in deficit schizophrenia: What do the dimensions of the Wisconsin Card Sorting Test tell us?. Schizophr. Res..

[15] Wang X., Yao S., Kirkpatrick B., Shi C., Yi J. (2008). Psychopathology and neuropsychological impairments in deficit and nondeficit schizophrenia of Chinese origin. Psychiatry Res..

[16] Tyburski E., Pełka-Wysiecka J., Mak M., Samochowiec A., Bieńkowski P., Samochowiec J. (2017). Neuropsychological Profile of Specific Executive Dysfunctions in Patients with Deficit and Non-deficit Schizophrenia. Front. Psychol..

[17] Cohen A.S., Docherty N.M. (2004). Deficit versus negative syndrome in schizophrenia: Prediction of attentional impairment. Schizophr. Bull..

[18] Cascella N.G., Testa S.M., Meyer S.M., Rao V.A., Diaz-Asper C.M., Pearlson G.D., Schretlen D.J. (2008). Neuropsychological impairment in deficit vs. non-deficit schizophrenia. J. Psychiatr. Res..

[19] Pegoraro L.F., Dantas C.R., Banzato C.E., Fuentes D. (2013). Correlation between insight dimensions and cognitive functions in patients with deficit and nondeficit schizophrenia. Schizophr. Res..

[20] Beck A.T., Grant P.M., Huh G.A., Perivoliotis D., Chang N.A. (2013). Dysfunctional attitudes and expectancies in deficit syndrome schizophrenia. Schizophr. Bull..

[21] Bryson G., Whelahan H.A., Bell M. (2001). Memory and executive function impairments in deficit syndrome schizophrenia. Psychiatry Res..

[22] Seckinger R.A., Goudsmit N., Coleman E., Harkavy-Friedman J., Yale S., Rosenfield P.J., Malaspina D. (2004). Olfactory identification and WAIS-R performance in deficit and nondeficit schizophrenia. Schizophr. Res..

[23] Chen C., Jiang W., Zhong N., Jiang H., Du J., Li Y., Ma X., Zhao M., Hashimoto K., Gao C. (2014). Impaired processing speed and attention in first-eIe drug naive schizophrenia with deficit syndrome. Schizophr. Res..

[24] Galderisi S., Maj M., Mucci A., Cassano G.B., Invernizzi G., Rossi A., Vita A., Dell’Osso L., Daneluzzo E., Pini S. (2002). Historical, psychopathological, neurological, and neuropsychological aspects of deficit schizophrenia: A multicenter study. Am. J. Psychiatry.

[25] Strauss E., Sherman E., Spreen O. (2006). A Compendium of Neuropsychological Tests: Administration, Norms, and Ommentary.

[26] Yu M., Tang X., Wang X., Zhang X., Zhang X., Sha W., Shu N., Zhang X.Y., Zhang Z. (2015). Neurocognitive impairments in deficit and non-deficit schizophrenia and their relationships with symptom dimensions and other clinical variables. PLoS ONE.

[27] Fervaha G., Agid O., Foussias G., Siddiqui I., Takeuchi H., Remington G. (2016). Neurocognitive impairment in the deficit subtype of schizophrenia. Eur. Arch. Psychiatry Clin. Neurosci..

[28] Bryson G., Bell M., Kaplan E., Greig T., Lysaker P. (1998). Affect recognition in deficit syndrome schizophrenia. Psychiatry Res..

[29] Strauss G.P., Jetha S.S., Ross S.A., Duke L.A., Allen D.N. (2010). Impaired faabellingect labeling and discrimination in patients with deficit syndrome schizophrenia. Schizophr. Res..

[30] Tang X.W., Yu M., Duan W.W., Zhang X.R., Sha W.W., Wang X., Zhang X.B. (2016). Facial emotion recognition and alexithymia in Chinese male patients with deficit schizophrenia. Psychiatry Res..

[31] Eack S.M., Greeno C.G., Pogue-Geile M.F., Newhill C.E., Hogarty G.E., Keshavan M.S. (2010). Assessing social-cognitive deficits in schizophrenia with the Mayer-Salovey-Caruso Emotional Intelligence Test. Schizophr. Bull..

[32] Harvey P.D., Koren D., Reichenberg A., Bowie C.R. (2006). Negative symptoms and cognitive deficits: What is the nature of their relationship?. Schizophr. Bull..

[33] Dibben C.R., Rice C., Laws K., McKenna P.J. (2009). Is executive impairment associated with schizophrenic syndromes? A meta-analysis. Psychol. Med..

[34] Nieuwenstein M.R., Aleman A., De Haan E.H. (2001). Relationship between symptom dimensions and neurocognitive functioning in schizophrenia: A meta-analysis of WCST and CPT studies. J. Psychiatr. Res..

[35] Henry J., Crawford J. (2005). A meta-analytic review of verbal fluency deficits in schizophrenia relative to other neurocognitive deficits. Cogn. Neuropsychiatry.

[36] Jędrasik-Styła M., Ciołkiewicz A., Styła R., Linke M., Parnowska D., Gruszka A., Denisiuk M., Jarema M., Green M.F., Wichniak A. (2015). The Polish academic version of the MATRICS Consensus Cognitive Battery (MCCB): Evaluation of psychometric properties. Psychiatr. Q..

[37] Nuechterlein K.H., Green M.F., Kern R.S., Baade L.E., Barch D.M., Cohen J.D., Essock S., Fenton W.S., Frese F.J., Gold J.M. (2008). The MATRICS Consensus Cognitive Battery, part 1: Test selection, reliability, and validity. Am. J. Psychiatry.

[38] Shafer A., Dazzi F. (2019). Meta-analysis of the positive and Negative Syndrome Scale (PANSS) factor structure. J. Psychiatr. Res..

[39] World Health Organization (WHO) (1993). The ICD-10 Classification of Mental and Behavioural Disorders.

[40] Sheehan D.V., Lecrubier Y., Sheehan K.H., Amorim P., Janavs J., Weiller E., Hergueta T., Baker R., Dunbar G.C. (1998). The Mini-International Neuropsychiatric Interview (M.I.N.I.): The development and validation of a structured diagnostic psychiatric interview for DSM-IV and ICD-10. J. Clin. Psychiatry.

[41] Podwalski P., Tyburski E., Szczygieł K., Waszczuk K., Rek-Owodziń K., Mak M., Plichta P., Bielecki M., Rudkowski K., Kucharska-Maalr J. (2021). White Matter Integrity of the Corpus Callosum and Psychopathological Dimensions in Deficit and Non-Deficit Schizophrenia Patients. J. Clin. Med..

[42] Brzeziński J., Gaul M., Hornowska E., Jaworowska A., Machowski A., Zakrzewska M. (2004). Wechsler Adult Intelli–Ence Scale-Revised. Polish Normalization.

[43] Khandaker G.M., Barnett J.H., White I.R., Jones P.B. (2011). A quantitative meta-analysis of population-based studies of premorbid intelligence and schizophrenia. Schizophr. Res..

[44] Missar C.D., Gold J.M., Goldberg T.E. (1994). WAIS-R short forms in chronic schizophrenia. Schizophr. Res..

[45] Russell A.J., Munro J., Jones P.B., Hayward P., Hemsley D.R., Murray R.M. (2000). The National Adult Reading Test as a measure of premorbid IQ in schizophrenia. Br. J. Clin. Psychol..

[46] Blyler C.R., Gold J.M., Iannone V.N., Buchanan R.W. (2000). Short form of the WAIS-III for use with patients with schizophrenia. Schizophr. Res..

[47] Miller H.R., Streiner D.L., Goldberg J.O. (1996). Short, shorter, shortest: The efficacy of WAIS-R short forms with mixed psychiatric patients. Assessment.

[48] Christensen B.K., Girard T.A., Bagby R.M. (2007). Wechsler Adult Intelligence Scale-short form for index and IQ scores in a psychiatric population. Psychol. Assess..

[49] Sumiyoshi C., Fujino H., Sumiyoshi T., Yasuda Y., Yamamori H., Ohi K., Fujimoto M., Takeda M., Hashimoto R. (2016). Usefulness of the Wechsler Intelligence Scale short form for assessing functional outcomes in patients with schizophrenia. Psychiatry Res..

[50] Bulzacka E., Meyers J.E., Boyer L., Le Gloahec T., Fond G., Szöke A., Leboyer M., Schürhoff F. (2016). WAIS-IV seven-subtest short form: Validity and clinical use in schizophrenia. Arch. Clin. Neuropsychol..

[51] Kay S.R., Fiszbein A., Opler L.A. (1987). The positive and negative syndrome scale (PANSS) for schizophrenia. Schizophr. Bull..

[52] Rzewuska M. (2002). Validity and reliability of the Polish version of the Positive and Negative Syndrome Scale (PANSS). Int. J. Methods Psychiatr. Res..

[53] Tatsumi K., Kirkpatrick B., Strauss G.P., Opler M. (2020). The Brief Negative Symptom Scale in Translation: A Review of Psychometric Properties and Beyond. Eur. Neuropsychopharmacol..

[54] Dollfus S., Mach C., Morello R. (2016). Self-Evaluation of Negative Symptoms. Schizophr. Bull..

[55] Hall R.C. (1995). Global Assessment of Functioning: A Modified Scale. Psychosomatics.

[56] Hair J.F., Black W.C., Babin B.J., Anderson R.E. (2010). Multivariate Data Analysis.

[57] Sakia R.M. (1992). The Box-Cox transformation technique: A review. J. R. Stat. Soc..

[58] Cohen J. (1992). A power primer. Psychol. Bull..

[59] Perugini M., Gallucci M., Costantini G. (2018). A practical primer to power analysis for simple experimental designs. Int. Rev. Soc. Psychol..

[60] Leucht S., Samara M., Heres S., Davis J.M. (2016). Dose equivalents for antipsychotic drugs: The DDD method. Schizophr. Bull..

[61] Buczylowska D., Petermann F. (2016). Age-related differences and heterogeneity in executive functions: Analysis of NAB executive functions module scores. Arch. Clin. Neuropsychol..

[62] Li W., Zhou F.C., Zhang L., Ng C.H., Ungvari G.S., Li J., Xiang Y.T. (2020). Comparison of cognitive dysfunction between schizophrenia and bipolar disorder patients: A meta-analysis of comparative studies. J. Affect. Disord..

[63] Tiryaki A., Yazıcı M.K., Anıl A.E., Kabakçı E., Karaağaoğlu E., Göğüş A. (2003). Reexamination of the characteristics of the deficit schizophrenia patients. Eur. Arch. Psychiatry Clin. Neurosci..

[64] Girard R., Météreau E., Thomas J., Pugeat M., Qu C., Dreher J.C. (2017). Hormone therapy at early post-menopause increases cognitive control-related prefrontal activity. Sci. Rep..

