# Supplementary material for: Cognitive Dysfunctions Measured with the MCCB in Deficit and Non-Deficit Schizophrenia
Source: J Clin Med. 2023 Mar 14;12(6):2257. doi: 10.3390/jcm12062257 (PMC10053076; doi:10.3390/jcm12062257)
Supplement: Supplementary file 1 [file jcm-12-02257-s001.zip › jcm-2168199-supplementary.pdf]

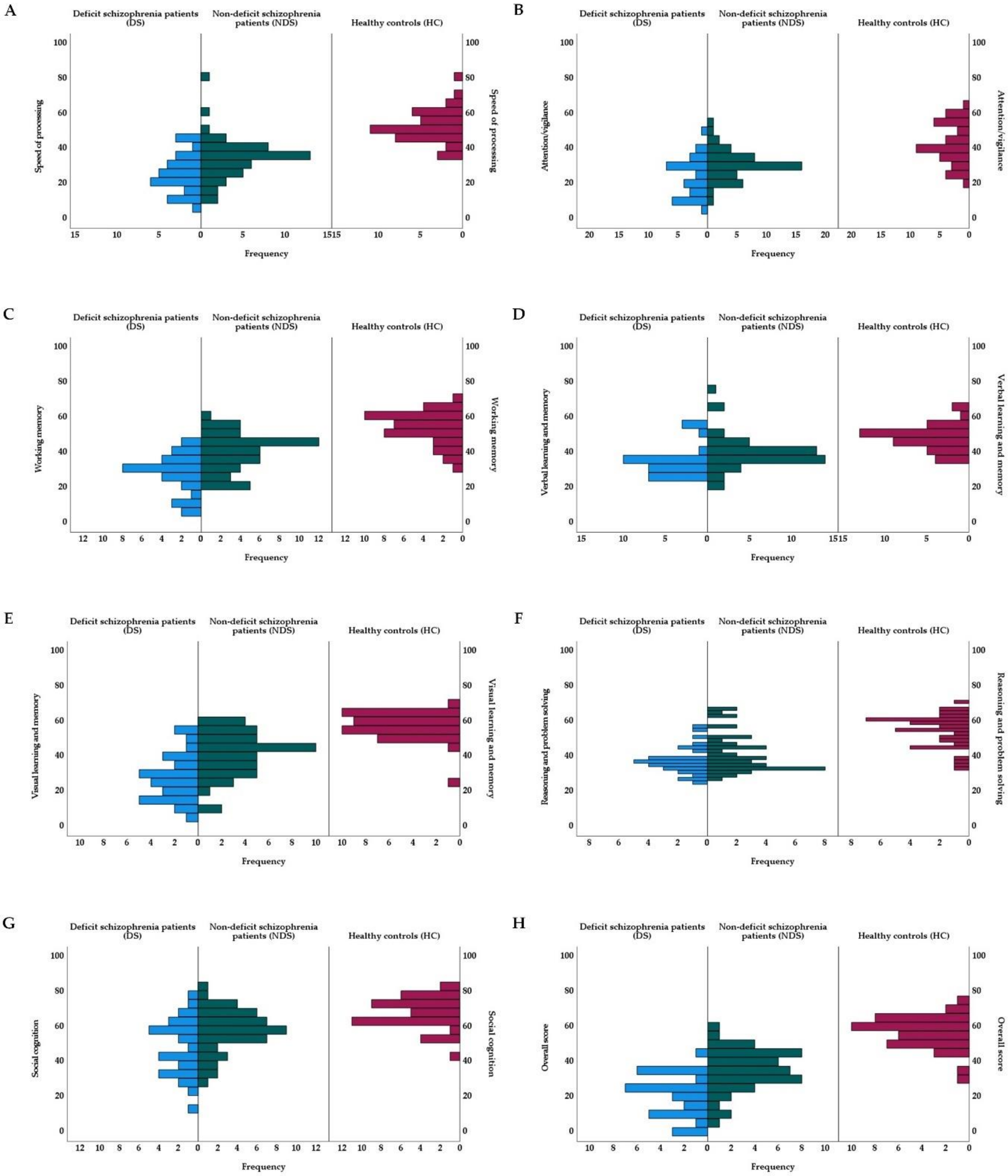

Figure S1. Distributions of the samples on cognitive functions from three groups – untransformed T-scores (MCCB: (**A**) = speed of processing; (**B**) = attention/vigilance; (**C**) = working memory; (**D**) = verbal learning and memory; (**E**) = visual learning and memory; (**F**) = reasoning and problem solving; (**G**) = social cognition, and (**H**) = overall score). MCCB = Measurement and Treatment Research to Improve Cognition in Schizophrenia.
